# Identification of heterogeneity among soft tissue sarcomas by gene expression profiles from different tumors

**DOI:** 10.1186/1479-5876-6-23

**Published:** 2008-05-06

**Authors:** Keith M Skubitz, Stefan Pambuccian, J Carlos Manivel, Amy PN Skubitz

**Affiliations:** 1 Department of Medicine, the University of Minnesota Medical School, Masonic Cancer Center, Minneapolis, MN 55455, USA; 2 Department of Laboratory Medicine and Pathology, the University of Minnesota Medical School, Masonic Cancer Center, Minneapolis, MN 55455, USA

## Abstract

The heterogeneity that soft tissue sarcomas (STS) exhibit in their clinical behavior, even within histological subtypes, complicates patient care. Histological appearance is determined by gene expression. Morphologic features are generally good predictors of biologic behavior, however, metastatic propensity, tumor growth, and response to chemotherapy may be determined by gene expression patterns that do not correlate well with morphology. One approach to identify heterogeneity is to search for genetic markers that correlate with differences in tumor behavior. Alternatively, subsets may be identified based on gene expression patterns alone, independent of knowledge of clinical outcome. We have reported gene expression patterns that distinguish two subgroups of clear cell renal carcinoma (ccRCC), and other gene expression patterns that distinguish heterogeneity of serous ovarian carcinoma (OVCA) and aggressive fibromatosis (AF). In this study, gene expression in 53 samples of STS and AF [including 16 malignant fibrous histiocytoma (MFH), 9 leiomyosarcoma, 12 liposarcoma, 4 synovial sarcoma, and 12 samples of AF] was determined at Gene Logic Inc. (Gaithersburg, MD) using Affymetrix GeneChip^® ^U_133 arrays containing approximately 40,000 genes/ESTs. Gene expression analysis was performed with the Gene Logic Genesis Enterprise System^® ^Software and Expressionist software. Hierarchical clustering of the STS using our three previously reported gene sets, each generated subgroups within the STS that for some subtypes correlated with histology, and also suggested the existence of subsets of MFH. All three gene sets also recognized the same two subsets of the fibromatosis samples that we had found in our earlier study of AF. These results suggest that these subgroups may have biological significance, and that these gene sets may be useful for sub-classification of STS. In addition, several genes that are targets of some anti-tumor drugs were found to be differentially expressed in particular subsets of STS.

## Introduction

Soft tissue sarcomas (STS) exhibit heterogeneity in their clinical behavior, even within the same histological subtypes. This heterogeneity is an important problem in the treatment of patients with STS. Gene expression is felt to play a critical role in cell development and malignant transformation, and gene expression profiles are of potential use in the classification and diagnosis of malignancies [1-3]. Several recent studies have found distinctive gene expression patterns that can differentiate between histologic subtypes of STS [4-10].

Histological appearance is determined by gene expression. However, metastatic propensity, tumor growth, and response to therapy may be determined by gene expression patterns that do not correlate well with morphology. One approach to identify heterogeneity is to search for markers that correlate with differences in tumor behavior. Alternatively, subsets may be identified based on gene expression patterns independent of knowledge of clinical outcome. We have reported gene expression patterns that distinguish two subsets of clear cell renal cell carcinoma (ccRCC) [11], two major subgroups of aggressive fibromatosis (AF) [12], and other patterns that distinguish borderline from invasive serous ovarian carcinoma (OVCA) [13].

In the current study, we sought to identify subsets of STS based on patterns of gene expression using these three gene sets. We also searched for the expression of genes that encode targets of selected therapies in different STS subsets. Gene expression levels were quantified by the use of microarray technology using the Affymetrix GeneChip^® ^U_133 microarray, representing ~40,000 known genes and expression sequence tags (ESTs). Hierarchical clustering of the STS using our three previously reported gene sets, each generated subgroups within the STS that for some, but not all, subtypes correlated with histology.

The term "malignant fibrous histiocytoma" (MFH) is currently used for pleomorphic sarcomas without identifiable defined differentiation [14]. However, MFH may not represent a distinct tumor entity; indeed many sarcomas previously termed MFH appear to share biochemical markers with other subtypes of STS, but are not readily recognized as such based on their histologic appearance. In this study, we also searched for potential subsets among cases that were diagnosed as MFH. In a set of 16 MFH samples, two major subsets were identified on the basis of gene expression.

We conclude that gene expression profiles may be useful in sub-classifying STS. In addition, differences in gene expression patterns may help identify potential therapies in STS.

## Materials and methods

### Tissue samples

Tissue from 53 soft tissue tumors, including 41 sarcomas and 12 AF (see additional file 1), were obtained from the University of Minnesota Cancer Center's Tissue Procurement Facility. Samples were obtained using protocols approved by the University of Minnesota Institutional Review Board. Tumor samples were identified by a pathologist, dissected ensuring the presence of gross tumor and the absence of necrosis, and snap frozen in liquid nitrogen within 30 min of excision. Parallel tissue sections of each sample were processed routinely, and were examined by light microscopy after H&E staining to confirm the pathologic nature of the sample. In addition, diagnoses were also confirmed by the Tissue Procurement Facility pathologist and by a separate review by a pathologist at Gene Logic Inc. (Gaithersburg, MD).

### Pathology review and grading

Slides of the STS and AF tumors from which samples had been obtained were reviewed independently by two pathologists experienced in the field of STS without knowledge of their gene expression profiles. All slides available from primary tumors and implants/metastases were reviewed, without knowledge of the original diagnosis or the clinical characteristics of the patients, and assigned a histologic type and grade. Histologic type was determined according to the WHO [15]. The mitotic rate was determined by counting mitoses in 50 high-power fields (HPF) and averaged mitotic counts for 10 HPF. Necrosis was estimated from both the gross description of the tumor and review of all available slides. Histologic grading was performed according to the National Cancer Institute (NCI) system proposed in 1984 by Costa [16] and the updated version of the European system (FNCLCC) [17]. In brief, the NCI system employs three grades based on a combination of histologic diagnosis, cellularity, cellular pleomorphism, mitotic activity and tumor necrosis. A variation of the Costa grading system (as described by Kandel et al [18]) was used, where a score of 1 was assigned to each of the following features: > 6 mitoses/10 HPF, presence of cellular pleomorphism, presence of high tumor cellularity (> 50% cells to matrix), and > 15% tumor necrosis. Tumors with a cumulative score of 1 were assigned grade 1, those with a score of 2 were assigned grade 2, and those with a score of >/= 3 were grade 3. The FNCLCC (Federation Nationale des Centres de Lutte Contre le Cancer) system also employs three grades. Briefly summarized, the FNCLCC system assigns a score to tumor differentiation (1: sarcoma closely resembling normal adult mesenchymal tissue; 2: sarcoma for which the histologic typing is certain; 3: embryonal and undifferentiated sarcomas; sarcomas of uncertain type), a score for mitotic activity (1: 0–9/10 HPF; 2: 10–19/10 HPF; 3: 20 or more/10 HPF), and a score for tumor necrosis (0: no tumor necrosis; 1: 50% or less necrosis; 2: > 50% necrosis). The histologic grade is 1 for total score 2 or 3; grade 2 for total score 4 or 5; and grade 3 for total score 6, 7 or 8. An attempt to simplify the grading into a two-tiered system of high and low grade tumors has also been proposed [18], and tumors were also subjectively classified into two grades (high or low) and into three grades (1,2,3) (see additional file 1).

### Gene expression analysis

RNA was prepared and gene expression was determined at Gene Logic Inc. using Affymetrix GeneChip^® ^U_133 microarrays containing oligodeoxynucleotides that correspond to approximately 40,000 genes/ESTs. Gene expression analysis was performed with the Gene Logic Genesis Enterprise System^® ^Software and Expressionist software as previously described [7-9,11-13,19]. Samples underwent stringent quality control measures in order to preserve the integrity of the RNA before use in gene microarray experiments. RNA was isolated by homogenization of frozen tissue in extraction buffer in RNase-free conditions. RNA quantity was determined spectrophotometrically, and the quality was assessed on agarose gels to confirm the presence of non-degraded RNA. Tissue samples were not used if the RNA yield was low or RNA degradation was evident. Biotinylated cRNAs were prepared using standard Affymetrix protocols. Briefly, RNA was converted to first strand cDNA followed by second strand synthesis. Double-stranded cDNA was used as the template for *in vitro *transcription using biotinylated ribonucleotides to generate biotin-labeled cRNA. Biotinylated cRNA was fragmented for target preparation and then hybridized on Affymetrix GeneChip^® ^U_133 microarrays according to the standard Affymetrix protocols. Following hybridization, the microarrays were washed and stained using an automated fluidics system. The microarrays were then digitally scanned and images of the average probe intensities were visually monitored for any irregularities in the microarrays. Samples were rehybridized when images appeared flawed in any way.

The integrity of the RNA sample was further monitored by examining the relative expression of a probe from the 3' end of beta-actin compared with the expression of a probe from the 5' end of the same gene. In addition, internal controls were provided on each Affymetrix microarray, and samples with "flawed" data were not utilized. The geometric means of the expression intensities of the relevant gene fragments were computed, and the ratio was reported as the fold change (up or down). Confidence intervals and p values on the fold change were calculated using a two-sided Welch modified two-sample t-test. Differences were considered significant when p </= 0.05. Principle Component Analysis (PCA), contrast analyses, and e-Northern^® ^analyses were performed using the Gene Logic Genesis Enterprise System^® ^Software. Clustering was performed using the Eisen clustering software and viewed using the Tree View software.

## Results

### Identification of AF subsets

**Figure 1:**
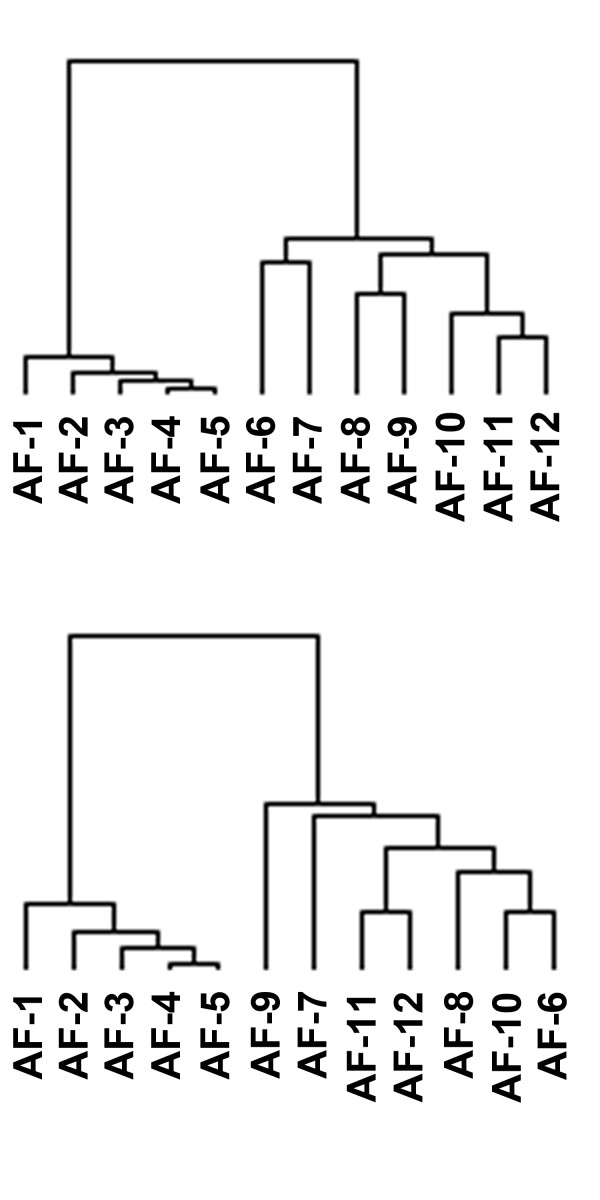
Clustering of gene expression in the aggressive fibromatosis samples using the RCC gene set (top) and OVCA gene set (bottom) and the Eisen clustering software Cluster. The 12 AF samples were clustered using the Eisen clustering software Cluster and the set of 167 gene fragments from the U_133 microarray set most differentially expressed between two groups of ccRCC previously described [11] (top) and the set of 200 gene fragments most differentially expressed between borderline and invasive OVCA [13] (bottom) as described in the text. Samples AF-1 to AF-5 formed a cluster, while samples AF-6 to AF-12 formed another cluster. The tissue samples in the tree are joined by very short branches if they have gene expression patterns that are very similar to each other, and by increasingly longer branches as their similarity decreases.

In a previous study we reported that a set of AF samples could be separated into two major subgroups, AF-1 to AF-5 (AF-set A) and AF-6 to AF-12 (AF-set B), as determined by gene expression patterns [12]. We identified a set of 200 gene fragments most differentially expressed between the two subsets of AF (AF gene set). We recently performed a similar series of gene expression analyses in a set of ccRCC, and identified two major subgroups of ccRCC [11]. Hierarchical clustering using the 167 gene fragments most differentially expressed between the two ccRCC subsets previously reported (RCC gene set) separated the AF samples into two major groups (Fig 1, top). In similar studies, we identified 200 gene fragments most differentially expressed between borderline and invasive OVCA (OVCA gene set) [13]. Similarly, hierarchical clustering of the AF samples using the OVCA gene set separated the AF samples into the same two major groups (Fig 1, bottom). Only 9 genes were present in both the RCC and OVCA gene sets, 16 genes in both the RCC and AF gene sets, and 4 genes in both the OVCA and AF gene sets (Table 1).

**Table 1:** Genes expressed in multiple gene sets

Gene Symbol	Gene Set		
	
	RCC^a^	OVCA^b^	AF^c^
CXCR4	X	X	
GPX3	X	X	
IgL	X	X	
LOC440871	X	X	
MT1G	X	X	
MT1H	X	X	
PRAME	X	X	
SERPINA1	X	X	
			
ACTN1	X		X
CD44	X		X
CKAP4	X		X
CSPG2	X		X
DCN	X		X
FABP5	X		X
FLNA	X		X
IGSF4	X		X
KDELR3	X		X
MT1E	X		X
MT1X	X		X
MT2A	X		X
PLOD2	X		X
TMEM45A	X		X
TUBA3	X		X
			
D6S1101, DST		X	X
IGFBP7		X	X
MEST		X	x
			
SULF1	X	X	X

^a^Gene set that distinguished two subsets of clear cell renal cell carcinoma (ref [11]).

^b^Gene set that distinguished borderline from invasive serous ovarian carcinoma (ref [13]).

^c^Gene set that defined two major subgroups of aggressive fibromatosis (ref [12]).

To better characterize the potential differences in expression of genes involved in specific signaling pathways and potential therapeutic targets, a series of gene sets were examined for differential expression between AF-set A (AF 1–5) and AF-set B (AF 6–12). First we examined a gene set for wnt-signaling, since differential expression in AF samples of some genes involved in wnt-signaling has been previously reported [12,20-27]. We found many genes involved in the wnt-signaling pathway to be differentially expressed in AF-set A compared with AF-set B. The genes that were up-regulated 4-fold or more in AF-set B are shown in Table 2. None were up-regulated in AF-set A.

**Table 2:** WNT pathway genes upregulated 4-fold or more in AF-set B compared to AF-set A

Gene Symbol	Gene Name	Fragment Name	Fold change-up in AF-set B
CCND2	cyclin D2	200953_s_at	7
CSNK1A1	casein kinase 1, alpha 1	208865_at	4.9
CSNK2B	casein kinase 2, beta polypeptide	201390_s_at	4.4
CTBP1	C-terminal binding protein 1	203392_s_at	7.8
CTBP2, LOC440008	C-terminal binding protein 2, LOC440008	210835_s_at	5.8
DVL3	dishevelled, dsh homolog 3 (Drosophila)	201908_at	6.4
FZD1	frizzled homolog 1 (Drosophila)	204451_at	4.8
JUN	v-jun sarcoma virus 17 oncogene homolog (avian)	201464_x_at	5.1
PPP2CB	protein phosphatase 2 (formerly 2A), catalytic subunit, beta isoform	201375_s_at	5.4
PPP3CB	protein phosphatase 3 (formerly 2B), catalytic subunit, beta isoform (calcineurin A beta)	202432_at	6.7
PRKACB	protein kinase, cAMP-dependent, catalytic, beta	202741_at	7.1
RAC1	ras-related C3 botulinum toxin substrate 1 (rho family, small GTP binding protein Rac1)	208640_at	5.4
RHOA	ras homolog gene family, member A	200059_s_at	5.6
RHOB	ras homolog gene family, member B	212099_at	8.2
SFRP2	secreted frizzled-related protein 2	223121_s_at	4.9
SFRP4	secreted frizzled-related protein 4	204051_s_at	5.6
SKP1A	S-phase kinase-associated protein 1A (p19A)	200718_s_at	5.9
TBL1XR1	transducin (beta)-like 1X-linked receptor 1	223013_at	4.8
WNT5A	wingless-type MMTV integration site family, member 5A	205990_s_at	6.9

Fold change analysis was performed using genes involved in the wnt-signaling pathway. Genes that were most differentially expressed between AF-set A and AF-set B are listed.

Next we compared the expression of genes encoding known growth factors, receptors, cytokines, proteins involved in the immune response, and signaling pathways, including angiogenesis and mTOR in AF-set A compared with AF-set B. The genes that were up-regulated 3-fold or more in AF-set B compared to AF-set A are listed in additional file 2. TGFB1I1, TGFB2, TGFB3, TGFBR1, TGFBR2, IGF1, IGFBP3, IGFBP4, IGFBP7 HDGF, PDGFC, PDGFRA, PDGFRB, HIF1A, EDNRA, CTGF, LEPR, and as previously reported, FAP and ADAM12, were all over-expressed in AF-set B compared with AF-set A. Genes involved in the initiation of translation were also differentially expressed in AF-set B compared with AF-set A.

### Identification of STS subsets

**Figure 2:**
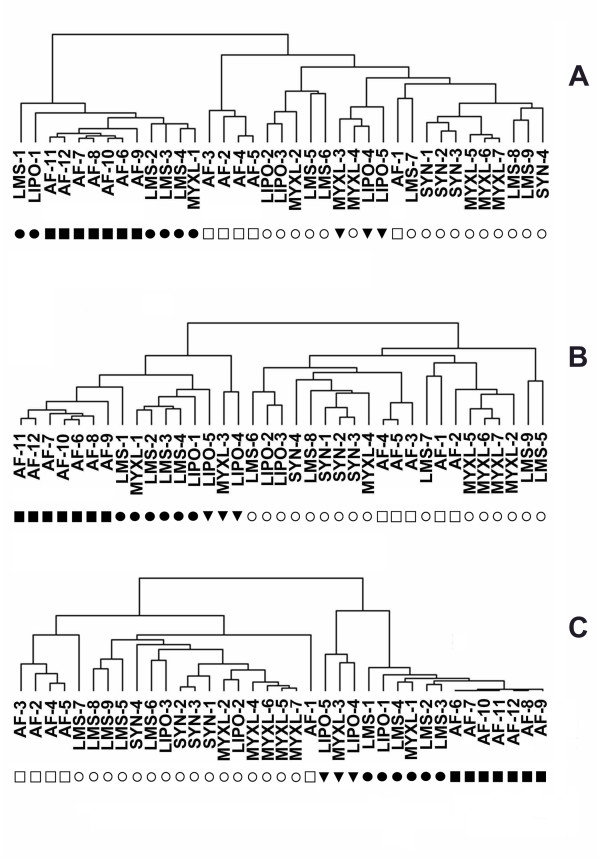
Clustering of gene expression of the STS and AF samples with the RCC gene set (A), OVCA gene set (B), and AF gene set (C). The 12 AF samples and the 25 other STS samples were clustered using the Eisen clustering software Cluster as described in the text. The 16 samples that cluster with AF-1 to AF-5 (open squares) using all 3 gene sets are indicated by open circles. The 6 samples that cluster with AF-6 to AF-12 (solid squares) using all 3 gene sets are indicated by closed circles. The clustering of 3 samples (solid triangles) varied with the gene set. The tissue samples in the tree are joined by very short branches if they have gene expression patterns that are very similar to each other, and by increasingly longer branches as their similarity decreases.

We questioned whether these same gene sets might distinguish subgroups of STS independent of histologic diagnosis. Hierarchical clustering was performed with the 12 AF samples, 4 synovial sarcomas, 7 myxoid liposarcomas, 5 other liposarcomas, and 9 leiomyosarcomas using the RCC gene set (Fig 2A), the OVCA gene set (Fig 2B), and the AF gene set (Fig 2C). Two major subgroups were observed in each case. With the RCC gene set (Fig 2A), one subgroup (solid circles and squares) contained 7 AF samples (AF-set B, i.e. AF-6 to AF-12) (solid squares), 4 leiomyosarcoma samples (solid circles), 1 myxoid liposarcoma (solid circle), and 1 round cell liposarcoma (solid circle). When hierarchical clustering was done using the OVCA gene set and the AF gene set (Fig 2B and 2C, respectively), this subgroup contained 3 additional samples (solid triangles) (1 round cell liposarcoma, 1 myxoid liposarcoma, and 1 other liposarcoma). The other major subgroup (open circles and squares), contained five AF samples (AF-set A, i.e. AF-1 to AF-5) (open squares), all four synovial sarcomas, the majority of the myxoid liposarcomas and leiomyosarcomas, and two other liposarcomas.

**Figure 3:**
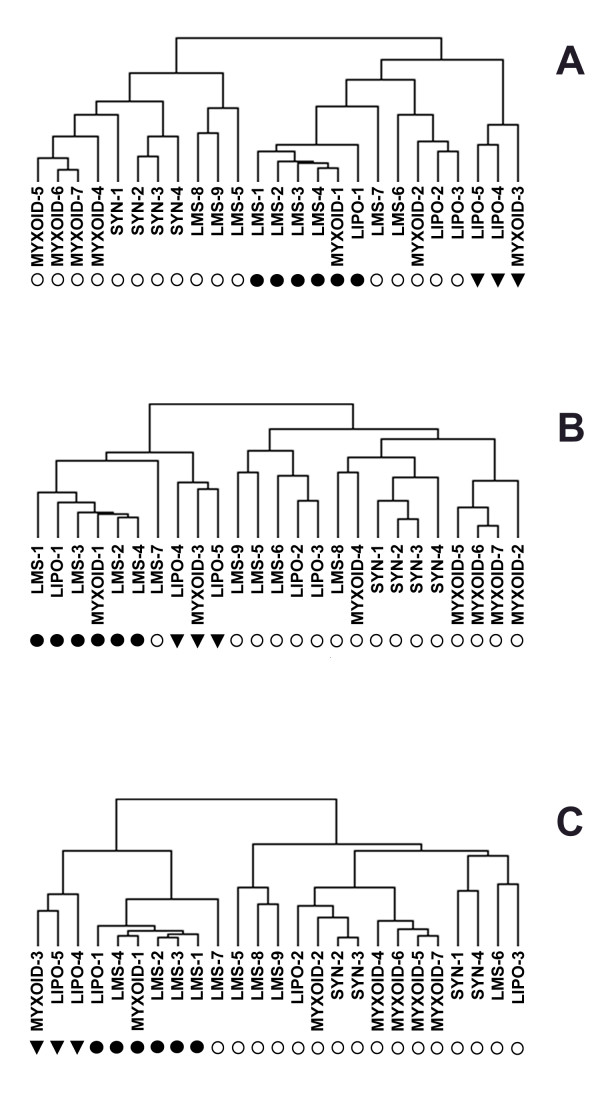
Clustering of gene expression of the STS samples with the RCC gene set (A), OVCA gene set (B), and AF gene set (C). The 25 STS samples were clustered using the Eisen clustering software Cluster as described in the text and are labeled as in Figure 2. The tissue samples in the tree are joined by very short branches if they have gene expression patterns that are very similar to each other, and by increasingly longer branches as their similarity decreases.

Since the results of hierarchical clustering are dependent on the composition of the sample sets, hierarchical clustering was then performed using the same three gene sets with the 25 STS samples, but without the AF samples (Fig 3). A similar clustering of the STS samples was again observed, whereby a subgroup consisting of the same four leiomyosarcoma samples, one myxoid liposarcoma, and one round cell liposarcoma (solid circles) clustered together, frequently with the three additional samples (solid triangles). Also, LMS-7 was now included in this cluster using all three gene sets.

**Figure 4:**
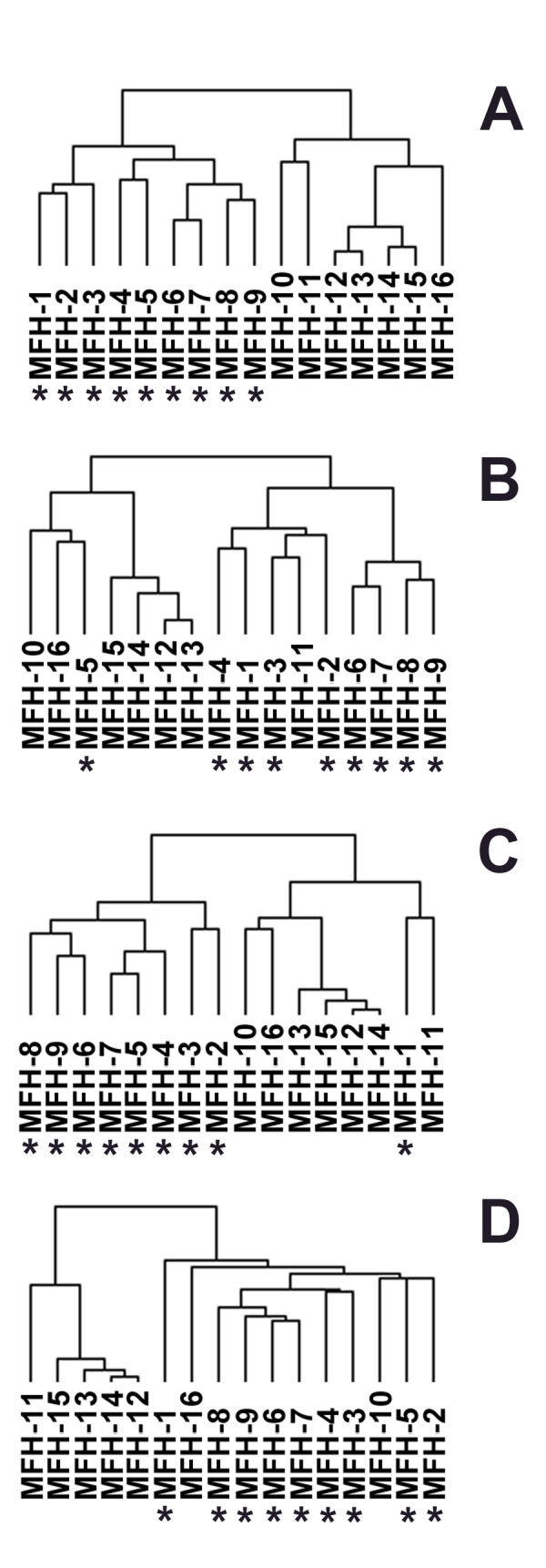
Clustering of gene expression of the MFH samples with the RCC gene set (A), OVCA gene set (B), AF gene set (C), and the protein kinase gene set (D) as described in the text. The 16 MFH samples were clustered using the Eisen clustering software Cluster as described in the text. MFH-1 to MFH-9 grouped together in panel A and are indicated by an asterisk. The tissue samples in the tree are joined by very short branches if they have gene expression patterns that are very similar to each other, and by increasingly longer branches as their similarity decreases.

Because MFH is not universally recognized as a diagnostic entity, and since STS with the diagnosis of MFH can have different biological behavior, we performed hierarchical clustering of the 16 MFH samples using the 3 gene sets. Hierarchical clustering using the RCC gene set generated two major subsets (Fig 4A), hereafter termed MFH-A (MFH-1 to MFH-9, indicated by an asterisk) and MFH-B (MFH-10 to MFH-16). Hierarchical clustering of the MFH samples using the OVCA gene set (Fig 4B), and the AF gene set (Fig 4C) also generated two clusters of the MFH samples similar to those described above. Since protein kinases are involved in many signaling pathways, hierarchical clustering of the MFH samples was performed using a set of 1500 probes that represent genes that encode protein tyrosine-, serine-, and threonine-kinases; these same two major clusters were again observed (Fig 4D). Each MFH subset appeared to contain a group of four MFH samples (MFH-12 to MFH-15) that were more closely clustered with each other than to the remaining samples of the subset. Because these subclusters contained only four samples, they were not further analyzed separately.

While more than two biologically distinct subsets of the 16 MFH samples likely exist, we focused our subsequent studies on differences between the two major subsets, MFH-A and MFH-B, defined above. A fold change analysis was performed comparing MFH-A to MFH-B. Those genes expressed at >/= 2-fold more between the two sets were then evaluated by e-Northern^® ^analysis. e-Northern^® ^analysis provides a graphical representation of the expression values for each sample. The genes most differentially expressed between MFH-A and MFH-B are listed in additional file 3. Interestingly, all of these genes were over-expressed in MFA-A compared with MFH-B, while none were over-expressed in MFH-B. Among the differentially expressed genes were genes that encode growth factors, receptors, and genes involved in various signaling pathways that are potential drug targets. For example, the 5.3-fold up-regulation of VEGF in MFH-A vs. MFH-B suggests that the efficacy of treatment directed against VEGF could differ in the two MFH subgroups. In addition, genes involved in the initiation of translation, another potential drug target, were also differentially expressed, (i.e. EIF1, EIF2B1, EIF2S, EIF3S7, EIF3S10, EIF4A1, EIF4B, EIF4G1, were all over-expressed > 2.6-fold in MFH-A compared to MFH-B).

Since heterogeneity of a drug target in a diagnostic category is critical to the outcome of a clinical trial, the expression of known potential target genes in the other types of STS was also examined. The expression of many of these genes, and genes for several tumor antigens that might be relevant in immunotherapy trials, was heterogeneous within diagnostic categories (Tables 3 and 4). For example, while VEGF (not shown) was expressed in all of the STS subtypes, TGFB1 and FGF7 (keratinocyte growth factor) were expressed in ~78% of LMS but only ~40% of non-myxoid liposarcoma, while OGFR was expressed in ~43% of myxoid liposarcoma, 100% of non-myxoid liposarcomas, ~67% of leiomyosarcomas, and none of the AF samples. Similarly, as regards the potential use of vaccines and immunotherapy, we observed differential expression of several genes. For example, CTAG2 was expressed in ~60% of the non-myxoid liposarcoma samples, but in none of the LMS, AF, or MFH samples. Also, PRAME was expressed in 100% of the non-myxoid liposarcomas and synovial sarcomas, but none of the AF or MFH-B samples.

**Table 3:** Differential Expression of potential target genes in STS samples

			Percent of Samples Expressing Gene				
			
Gene Symbol	Gene Name	Fragment Name	AF	Liposarcoma	LMS	Myxoid Liposarcoma	MFH
CTGF	connective tissue growth factor	209101_at	100	80	100	100	9
EGF	epidermal growth factor	206254_at	8	0	33	0	19
EGFR	epidermal growth factor receptor	232541_at	83	60	67	86	75
FAP	Fibroblast activation protein, alpha	209955_s_at	100	60	78	29	100
FGF13	fibroblast growth factor 13	205110_s_at	67	80	67	71	56
FGF18	fibroblast growth factor 18	231382_at	33	60	0	71	19
FGF2	fibroblast growth factor 2 (basic)	204422_s_at	33	40	33	14	31
FGF7	fibroblast growth factor 7 (keratinocyte growth factor)	205782_at	58	40	78	57	56
FGFR1	fibroblast growth factor receptor 1	222164_at	92	80	89	86	94
FGFR2	fibroblast growth factor receptor 2	208228_s_at	83	40	89	71	50
FLT1	fms-related tyrosine kinase 1 (vascular endothelial growth factor receptor)	226497_s_at	100	100	78	100	100
IGF1	insulin-like growth factor 1 (somatomedin C)	209541_at	100	100	67	100	94
IGF2R	insulin-like growth factor 2 receptor	201392_s_at	67	100	67	43	75
MET	met proto-oncogene (hepatocyte growth factor receptor)	203510_at	58	60	11	29	75
NGFB	nerve growth factor, beta polypeptide	206814_at	25	0	11	14	6
OGFR	opioid growth factor receptor	202841_x_at	0	100	67	43	75
PDGFA	platelet-derived growth factor alpha polypeptide	229830_at	17	0	11	0	13
PDGFB	platelet-derived growth factor beta polypeptide	216061_x_at	42	80	67	86	63
PDGFRA	platelet-derived growth factor receptor, alpha polypeptide	203131_at	100	100	67	100	100
PDGFRB	platelet-derived growth factor receptor, beta polypeptide	202273_at	100	100	89	100	100
PGF	placental growth factor, vascular endothelial growth factor-related protein	215179_x_at	100	100	100	100	100
TGFA	transforming growth factor, alpha	205016_at	8	0	22	14	63
TGFB1	transforming growth factor, beta 1	203085_s_at	83	40	78	57	100
TGFB2	transforming growth factor, beta 2	228121_at	92	60	67	86	81
TGFB3	transforming growth factor, beta 3	209747_at	100	40	89	43	88

**Table 4:** Differential expression of potential immunotherapy target genes in STS samples

		Percent of Samples Expressing Gene						
		
Gene Symbol	Gene Name	AF	Liposarcoma	LMS	Myxoid Liposarcoma	Synovial	MFH-B	MFH-A
CTAG2	cancer/testis antigen 2	0	60	0	71	75	0	0
CTAGE5	CTAGE family, member 5	75	60	44	29	25	71	67
MAGEA9	melanoma antigen family A, 9	0	40	111	71	75	14	0
MAGED1	melanoma antigen family D, 1	100	100	100	100	100	100	100
MAGED2	melanoma antigen family D, 2	100	80	89	100	50	86	78
MAGED4	melanoma antigen family D, 4	92	80	78	57	100	57	44
MAGEF1	melanoma antigen family F, 1	92	100	100	100	75	86	78
MAGEH1	melanoma antigen family H, 1	92	100	100	71	100	86	100
PRAME	preferentially expressed antigen in melanoma	0	100	33	86	100	0	11

Taken together, these data suggest that the AF and MFH sample sets can each be divided into two major subsets. Review of the pathology specimens was performed independently by two pathologists (Table 1), without knowledge of the clustering results. In all but 3 cases of the 40 non-AF cases, both pathologists agreed on high vs low grade. No clear histologic features were identified that correlated with the clusters described above, although the average mitosis score appeared lower in the MFH-A than in MFH-B set.

## Discussion

STS form a heterogeneous group of malignancies comprised of more than fifty distinct diagnostic categories [28-33]. Histologic criteria are useful in predicting outcome in STS. For example, in a comparative study of the NCI and FNCLCC grading systems in 410 adult patients with non-metastatic STS [17], both systems were of prognostic value for predicting metastases and overall survival, by univariate analysis. By multivariate analysis, high tumor grade, regardless of the system used, large tumor size (10 or more cm), and deep location, had independent prognostic value. Importantly, however, STS often exhibit heterogeneity of biological behavior even within diagnostic categories. This heterogeneity makes the clinical care of patients with these diseases challenging, and may also confound the development of drugs to treat these diseases. Thus, there is a need to move past simple histologic examination in STS trials.

The classification of STS has traditionally been determined by light microscopic examination of H&E stained tissues, in which recognizable characteristics are identified in the tumors, and more recently also by the use of genetic techniques (reviewed in [28-33]). Classification of tumors by gene expression profiles has the potential to provide additional useful information that is free of observer bias and variability, and aid in tumor classification and diagnosis. Analysis of gene expression by microarray also has the potential to identify heterogeneity among STS [4-6,8-10]. While one approach is to search for genes that correlate with clinical outcome, heterogeneity of the sample sets may complicate this search, as predictive genes may differ in different types of tumors. The search for genes that predict a particular behavior may also be complicated by genes whose expression is not relevant to the question of interest. Restriction of the number of genes in an analysis by elimination of irrelevant signals may help, if possible. In addition, relevant signals may or may not be different in different histological categories.

In the current report, potential subsets of STS were identified by gene expression profiles independent of knowledge of biological behavior using gene sets that differed between two subgroups of ccRCC, OVCA, and AF. The current report confirms that gene expression patterns can be used to identify subsets of STS directly, without searching for differences based on clinical correlates. This approach may also allow the identification of potential subsets that could be obscured by searching for patterns that discriminate between two predefined groups determined by a particular clinical outcome. Given the simplicity of the technology, there may be no need to identify the smallest gene set that can be used as a reproducible prognostic factor. Indeed, prognostic information may be lost by such an approach. The differences in gene expression observed between subgroups may reflect intrinsic differences in the tumor cells, differences in the host response to the tumor, or both. It is possible that these differences in gene expression patterns reflect differences in biology of these tumors, although we do not have adequate clinical outcome data to confirm this possibility. It is important to note that hierarchical clustering of any random set of samples using a random gene set may yield what appears to be two major clusters; further studies will be required to determine the reproducibility of the current findings and their potential practical utility.

Our findings, though limited by small sample size, suggest the existence of distinct subgroups within the MFH set. As tumors evolve, sequential mutation and/or epigenetic changes may result in increasing divergence in gene expression and biology from the original tumor. We previously reported that hierarchical clustering of gene expression patterns appeared to cluster some MFH samples near samples of liposarcoma, while others appeared to form more distinct clusters [9]. It is possible that analysis of a larger number of samples will identify additional clinically and biologically relevant subsets of MFH.

The current report also demonstrates a wide range among STS subgroups in the expression of genes that code for targets of some therapies. For example, PRAME, CTAG1, and CTAG2 have been reported to be over-expressed in liposarcoma compared with a variety of normal tissues [7], and recent studies have reported the expression of these and other CTAGs in several sarcoma subtypes [34,35]. The current results confirmed our earlier report of over-expression of PRAME, CTAG1 and CTAG2 in liposarcomas using the U_95 array [7] with the newer U_133 array, and also demonstrated that the expression of these potential targets of immunotherapy is heterogeneous among STS. Thus, studies in STS of agents with known targets would be strengthened by stratification by expression of target genes.

Notably, the three earlier studies that suggested the existence of two major subsets of AF, RCC, and OVCA, from which the gene sets used herein were derived, found differences in the expression of many extracellular matrix (ECM) genes between the respective subsets in each of the three diseases. This could be important for several reasons. First, the interaction of tumor cells with ECM proteins can have profound effects on cell biology, regulating signal transduction, apoptosis/anoikis, morphology, and tissue architecture. In this regard, expression of the ECM protein TGFBI has recently been reported to influence paclitaxel sensitivity of ovarian carcinoma cells [36]. ECM proteins can regulate interactions between growth factors and ovarian hormones in mammary epithelial cells, and laminin inhibits estrogen-induced proliferation of breast cancer cells [37]. Interactions with ECM components have been reported to alter TNF-alpha induced changes in endothelial permeability [38], and the ECM proteoglycan decorin can inhibit growth of pancreatic carcinoma cells [39]. Similarly, fibrin clots can promote motility of fibroblasts and endothelial cells, and fibrinogen degradation products may be angiogenic [40,41]. Second, the response of the host to the tumor may also play an important role in tumor biology, and ECM expression, by either tumor cells or host stromal cells, could reflect the local host response. For example, stromal fibroblasts could produce factors that alter tumor cell growth/biology [42].

It would be reasonable to stratify patients entering clinical trials using a technique similar to that described herein, in an attempt to decrease the problem of heterogeneity of the study population. Even if not performed in real time, tumor samples should be saved for a later analysis of this type. Since the results of a clustering analysis depend upon the composition of the sample set analyzed, such an approach would use a standard reference group of STS samples with clustering performed with the addition of the new test sample.

When comparing histologic grading of soft tissue sarcomas and their gene expression, it is important to take into consideration several facts. Histologic grading is an imperfect exercise; when the reproducibility of the European system was tested by 15 pathologists [43], an agreement was reached in 81% of the cases for tumor necrosis, 74% for tumor differentiation, 73% for mitotic rate, and 75% for overall tumor grade, although the agreement for histologic type was only 61%. In a study in which the NCI and FNCLCC grading systems were compared, there were discrepancies in ~35% of the cases [17]. Compared to the NCI system, the FNCLCC system produced a greater number of grade 3 tumors, a lower number of grade 2 tumors, and had better correlation with metastases-free and overall survival. Despite the limitations in reproducibility, numerous studies have confirmed the prognostic value of histologic grading of soft tissue sarcomas [44-46].

Any new technique that attempts to establish new pathways for prognostication should be compared to currently available techniques, and its superiority should be documented. Gene expression profiles, in addition to providing potentially useful prognostic information, may yield insight into two important aspects of sarcoma; namely, the identification of therapeutic targets, leading to more individualized therapies, and second, better understanding of the genesis, progression, and biology of these tumors.

The current study supports the use of gene expression patterns as a complementary set of data that may augment the use of light microscopy to help classify STS. Analysis of a larger number of samples and correlation of biological phenotypes with gene expression patterns may identify clinically meaningful characteristics of the subsets identified herein.

## Competing interests

The authors declare that they have no competing interests.

## Authors' contributions

KMS participated in study design, data analysis, and helped draft the manuscript

SP performed the pathologic examination, and helped analyze data and draft the manuscript

JCM performed the pathologic examination, and helped analyze data and draft the manuscript

APNS helped design the study, analyze data, and draft the manuscript

## Supplementary Material

Additional file 1 Samples. Pathologic description of the samples. Click here for file

Additional file 2 Genes encoding growth factors, receptors, cytokines, and proteins involved in the immune response and signaling pathways upregulated 3-fold or more in AF-set B compared to AF-set A. Genes encoding known growth factors, receptors, cytokines, and proteins involved in the immune response and signaling pathways including angiogenesis and mTOR, that were most differentially expressed between AF-set A and AF-set B are listed. Click here for file

Additional file 3 Genes upregulated 3-fold or more in MFH-A compared to MFH-B. Genes that were most differentially expressed between MFH-A and MFH-B were selected by e-Northern^® ^analysis and are listed. Click here for file
