# Supplementary material for: Identification of heterogeneity among soft tissue sarcomas by gene expression profiles from different tumors
Source: J Transl Med. 2008 May 6;6:23. doi: 10.1186/1479-5876-6-23 (PMC2412854; doi:10.1186/1479-5876-6-23)
Supplement: Additional file 1 — Samples. Pathologic description of the samples. [file 1479-5876-6-23-S1.doc]

Additional file 1. Samples*

| Sample | FNCLCC | | | | NCI | Subjective | | Age/  sex | Diagnosis |
| --- | --- | --- | --- | --- | --- | --- | --- | --- | --- |
|  | Differentiation | Mitosis | Necrosis | grade(sum) |  | Low/High | Grade |  |  |
| AF-1 |  |  |  |  |  |  |  | 36 M | AF |
| AF-2 |  |  |  |  |  |  |  | 68 F | AF |
| AF-3 |  |  |  |  |  |  |  | 23 M | AF |
| AF-4 |  |  |  |  |  |  |  | 35 F | AF |
| AF-5 |  |  |  |  |  |  |  | 13 M | AF |
| AF-6 |  |  |  |  |  |  |  | 33 F | AF |
| AF-7 |  |  |  |  |  |  |  | 16 M | AF |
| AF-8 |  |  |  |  |  |  |  | 21 M | AF |
| AF-9 |  |  |  |  |  |  |  | 22 F | AF |
| AF-10 |  |  |  |  |  |  |  | 33 F | AF |
| AF-11 |  |  |  |  |  |  |  | 23 M | AF |
| AF-12 |  |  |  |  |  |  |  | 19 M | AF |
| MFH-1 | 2 | 3 | 1 | 3 (6) | 3 | H | 3 | 91 F | MFH |
|  | 3 | 3 | 1 | 3 (7) | 3 | H | 3 |  | MFH |
| MFH-2 | 2 | 2 | 1 | 2 (5) | 3 | H | 3 | 40 M | MFH |
|  | 3 | 2 | 1 | 3 (6) | 3 | H | 3 |  | MFH |
| MFH-3 | 2 | 3 | 1 | 3 (6) | 3 | H | 3 | 67 F | MFH |
|  | 3 | 3 | 1 | 3 (7) | 3 | H | 3 |  | MFH |
| MFH-4 | 2 | 3 | 1 | 3 (6) | 2 | H | 3 | 86 M | Myxoid MFH |
|  | 2 | 3 | 1 | 3 (6) | 3 | H | 3 |  | MFH |
| MFH-5 | 2 | 1 | 1 | 2 (4) | 3 | H | 3 | 74 M | MFH |
|  | 2 | 1 | 1 | 2 (4) | 2 | H | 3 |  | MFH |
| MFH-6 | 3 | 1 | 1 | 2 (5) | 3 | H | 3 | 61 M | MFH |
|  | 3 | 1 | 1 | 2 (5) | 3 | H | 3 |  | MFH |
| MFH-7 | 3 | 1 | 2 | 3 (6) | 3 | H | 3 | 56 F | MFH |
|  | 3 | 1 | 2 | 3 (6) | 3 | H | 3 |  | MFH |
| MFH-8 | 3 | 2 | 1 | 3 (6) | 3 | H | 3 | 90 F | MFH |
|  | 3 | 3 | 1 | 3 (7) | 3 | H | 3 |  | MFH |
| MFH-9 | 3 | 1 | 1 | 2 (5) | 3 | H | 3 | 77 F | MFH |
|  | 3 | 2 | 2 | 3 (7) | 3 | H | 3 |  | MFH |
| MFH-10 |  |  |  |  |  |  |  |  | not available for review |
| MFH-11 | 2 | 3 | 2 | 3 (7) | 3 | H | 3 | 42 F | MFH |
|  | 3 | 3 | 1 | 3 (7) | 3 | H | 3 |  | MFH |
| MFH-12 | 2 | 3 | 1 | 3 (6) | 3 | H | 3 | 50 M | MFH |
|  | 3 | 3 | 1 | 3 (7) | 3 | H | 3 |  | MFH |
| MFH-13 | 2 | 3 | 2 | 3 (7) | 3 | H | 3 | 47 M | MFH |
|  | 3 | 3 | 3 | 3 (9) | 3 | H | 3 |  | MFH |
| MFH-14 | 3 | 3 | 1 | 3 (7) | 3 | H | 3 | 59 F | MFH |
|  | 3 | 3 | 1 | 3 (7) | 3 | H | 3 |  | MFH |
| MFH-15 | 3 | 2 | 1 | 2 (6) | 3 | H | 3 | 44 M | MFH |
|  | 3 | 3 | 2 | 3 (8) | 3 | H | 3 |  | MFH |
| MFH-16 | 2 | 1 | 1 | 2 (4) | 3 | H | 3 | 69 F | MFH |
|  | 3 | 3 | 1 | 3 (7) | 3 | H | 3 |  | MFH |
| LMS-1 | 2 | 1 | 1 | 2 (4) | 3 | H | 3 | 52 M | LMS |
|  | 2 | 1 | 1 | 2 (4) | 2 | H | 3 |  | LMS |
| LMS-2 | 3 | 2 | 1 | 3 (6) | 3 | H | 3 | 60 F | LMS |
|  | 3 | 3 | 1 | 3 (7) | 3 | H | 3 |  | LMS |
| LMS-3 | 2 | 2 | 1 | 2 (5) | 2 | H | 2 | 51 F | LMS |
|  | 1 | 1 | 0 | 1 (2) | 1 | L | 1 |  | LMS |
| LMS-4 | 3 | 1 | 1 | 2 (5) | 3 | H | 3 | 52 F | dedifferentiated LMS |
|  | 3 | 2 | 1 | 3 (6) | 3 | H | 3 |  | LMS |
| LMS-5 | 2 | 3 | 2 | 3 (7) | 3 | H | 3 | 61 F | LMS |
|  | 2 | 1 | 1 | 2 (4) | 2 | H | 2 |  | LMS |
| LMS-6 | 3 | 3 | 1 | 3 (7) | 3 | H | 3 | 52 F | LMS |
|  | 3 | 3 | 1 | 3 (7) | 3 | H | 3 |  | LMS |
| LMS-7 | 2 | 1 | 0 | 1 (3) | 2 | H | 2 | 57 F | LMS |
|  | 1 | 1 | 0 | 1 (2) | 1 | L | 1 |  | LMS |
| LMS-8 | 2 | 1 | 1 | 2 (4) | 3 | H | 3 | 73 F | LMS |
|  | 2 | 3 | 1 | 3 (6) | 3 | H | 3 |  | LMS |
| LMS-9 | 2 | 3 | 1 | 3 (6) | 3 | H | 3 | 55 F | LMS |
|  | 1 | 3 | 1 | 2 (5) | 3 | H | 2 |  | LMS |
| MYXOID-1 | 2 | 1 | 0 | 1 (3) | 1 | L | 1 | 28 F | Myxoid liposarcoma |
|  | 2 | 0 | 0 | 1 (3) | 1 | L | 1 |  | Myxoid liposarcoma |
| MYXOID-2 | 3 | 2 | 1 | 3 (6) | 3 | H | 3 | 62F | Liposarcoma myxoid and dedifferentiated |
|  | 2 | 1 | 0 | 1 (3) | 3 | H | 3 |  | Dedifferentiated liposarcoma |
| MYXOID-3 | 2 | 1 | 1 | 2 (4) | 2 | L | 2 | 40 M | Myxoid liposarcoma |
|  | 2 | 0 | 0 | 1 (2) | 1 | L | 1 |  | Myxoid liposarcoma |
| MYXOID-4 | 3 | 1 | 2 | 3 (6) | 3 | H | 3 | 45 M | LPS myxoid, round cell |
|  | 2 | 0 | 2 | 2 (4) | 1 | L | 1 |  | Myxoid liposarcoma |
| MYXOID-5 | 2 | 1 | 0 | 1 (3) | 1 | L | 1 | 38 M | Myxoid liposarcoma |
|  | 2 | 0 | 0 | 1 (2) | 1 | L | 1 |  | Myxoid liposarcoma |
| MYXOID-6 | 2 | 1 | 1 | 2 (4) | 1 | L | 1 | 43 F | Myxoid liposarcoma |
|  | 2 | 0 | 0 | 1 (2) | 1 | L | 1 |  | Myxoid liposarcoma |
| MYXOID-7 | 3 | 1 | 1 | 2 (5) | 3 | H | 3 | 74 M | Dedifferentiated liposarcoma |
|  | 3 | 3 | 0 | 3 (6) | 3 | H | 2 |  | Dedifferentiated liposarcoma |
| LIPO-1 | 3 | 3 | 2 | 3 (8) | 3 | H | 3 | 46 M | Round cell liposarcoma |
|  | 3 | 2 | 1 | 3 (6) | 3 | H | 3 |  | Round cell liposarcoma |
| LIPO-2 | 3 | 1 | 1 | 2 (5) | 3 | H | 3 | 86 F | MFH dediferentiated liposarcoma |
|  | 3 | 2 | 1 | 3 (6) | 3 | H | 3 |  | Pleomorphic liposarcoma |
| LIPO-3 | 2 | 3 | 1 | 3 (6) | 3 | H | 3 | 78 M | Dedifferentiated liposarcoma |
|  | 3 | 1 | 1 | 2 (5) | 3 | H | 3 |  | Dedifferentiated liposarcoma |
| LIPO-4 | 3 | 1 | 1 | 2 (5) | 3 | H | 3 | 50 M | Round cell liposarcoma |
|  | 3 | 2 | 1 | 3 (6) | 3 | H | 3 |  | Round cell liposarcoma |
| LIPO-5 | 3 | 1 | 1 | 2 (5) | 3 | H | 3 | 50 F | Round cell liposarcoma |
|  | 3 | 3 | 0 | 3 (6) | 3 | H | 3 |  | Round cell liposarcoma |
| SYN-1 | 3 | 2 | 0 | 2 (5) | 3 | H | 3 | 19 F | Synovial sarcoma |
|  | 3 | 3 | 0 | 3 (6) | 3 | H | 3 |  | Synovial sarcoma |
| SYN-2 | 3 | 1 | 0 | 2 (6) | 3 | H | 3 | 68 M | Synovial sarcoma |
|  | 3 | 1 | 0 | 2 (4) | 3 | H | 3 |  | Synovial sarcoma |
| SYN-3 | 3 | 1 | 0 | 2 (4) | 3 | H | 3 | 15 M | Synovial sarcoma |
|  | 2 | 1 | 0 | 1 (3) | 3 | H | 3 |  | Synovial sarcoma |
| SYN-4 | 3 | 1 | 1 | 2 (5) | 3 | H | 3 | 57 M | Synovial sarcoma |
|  | 3 | 2 | 1 | 3 (6) | 3 | H | 3 |  | Synovial sarcoma |

*Specimens were reviewed by two pathologists experienced in the field of sarcoma. The first row for each sample shows the interpretation of one pathologist, and the second row the interpretation of the second pathologist. Both pathologists concurred on each of the AF samples so only one interpretation is shown for these samples. MFH-10 was not available for review. Both FNCLCC and NIH systems were used. For FNCLCC, values are shown for tumor differentiation, mitotic activity, tumor necrosis, and FNCLCC grade (with sum score in parenthesis). Subjective classification as low or high, and the subjective grade are also shown.
