# Supplementary material for: Identification of heterogeneity among soft tissue sarcomas by gene expression profiles from different tumors
Source: J Transl Med. 2008 May 6;6:23. doi: 10.1186/1479-5876-6-23 (PMC2412854; doi:10.1186/1479-5876-6-23)
Supplement: Additional file 2 — Genes encoding growth factors, receptors, cytokines, and proteins involved in the immune response and signaling pathways upregulated 3-fold or more in AF-set B compared to AF-set A. Genes encoding known growth factors, receptors, cytokines, and proteins involved in the immune response and signaling pathways including angiogenesis and mTOR, that were most differentially expressed between AF-set A and AF-set B are listed. [file 1479-5876-6-23-S2.doc]

Additional file 2. Genes encoding growth factors, receptors, cytokines, and proteins involved in the immune response and signaling pathways upregulated 3-fold or more in AF-set B compared to AF-set A

| Gene Symbol | Gene Name | Fragment Name | Fold change up in AF-set B |
| --- | --- | --- | --- |
| ADAM12 | ADAM metallopeptidase domain 12 (meltrin alpha) | 202952_s_at | 9.7 |
| ANTXR1 | anthrax toxin receptor 1 | 224694_at | 4.4 |
| ANTXR2 | anthrax toxin receptor 2 | 225524_at | 5.8 |
| ARTS-1, CAST | (calpastatin, type 1 tumor necrosis factor receptor shedding aminopeptidase regulator) | 212586_at | 5.5 |
| BZRP | benzodiazapine receptor (peripheral) | 202096_s_at | 4.7 |
| C1QR1 | complement component 1, q subcomponent, receptor 1 | 202878_s_at | 6.6 |
| CTGF | connective tissue growth factor | 209101_at | 4.2 |
| D2B, PTPRG, RPL10A | (inhibitor of DNA binding 2B, dominant negative helix-loop-helix protein, protein tyrosine phosphatase, receptor type, G, ribosomal protein L10a) | 200036_s_at | 3.9 |
| DBI | diazepam binding inhibitor (GABA receptor modulator, acyl-Coenzyme A binding protein) | 202428_x_at | 5.8 |
| DCN, SSR1 | (decorin, signal sequence receptor, alpha (translocon-associated protein alpha)) | 201894_s_at | 6.5 |
| DDI1, PDGFD | (DNA-damage inducible protein 1, platelet derived growth factor D) | 219304_s_at | 4.5 |
| DDR2 | discoidin domain receptor family, member 2 | 225442_at | 5.3 |
| EDG2 | endothelial differentiation, lysophosphatidic acid G-protein-coupled receptor, 2 | 204036_at | 9.6 |
| EDNRA | endothelin receptor type A | 204464_s_at | 7.6 |
| EIF1 | eukaryotic translation initiation factor 1 | 211956_s_at | 3.5 |
| EIF2S2 | eukaryotic translation initiation factor 2, subunit 2 beta, 38kDa | 208726_s_at | 3.8 |
| EIF4A1 | eukaryotic translation initiation factor 4A, isoform 1 | 211787_s_at | 4.8 |
| EIF4A2, RNU107 | (RNA, U107 small nucleolar, eukaryotic translation initiation factor 4A, isoform 2) | 200912_s_at | 4 |
| EIF4B | eukaryotic translation initiation factor 4B | 211938_at | 4.3 |
| EIF4G2, LOC144017 | (eukaryotic translation initiation factor 4 gamma, 2, hypothetical protein LOC144017) | 200004_at | 4.6 |
| ELAVL1 | ELAV (embryonic lethal, abnormal vision, Drosophila)-like 1 (Hu antigen R) | 201726_at | 6.7 |
| F2RL2 | coagulation factor II (thrombin) receptor-like 2 | 230147_at | 6 |
| FAP | fibroblast activation protein, alpha | 209955_s_at | 5.91 |
| FKBP1A | FK506 binding protein 1A, 12kDa | 200709_at | 6.8 |
| FOS | v-fos FBJ murine osteosarcoma viral oncogene homolog | 209189_at | 7.4 |
| GABARAP | GABA(A) receptor-associated protein | 200645_at | 5.3 |
| GABARAPL2 | GABA(A) receptor-associated protein-like 2 | 209046_s_at | 8 |
| HDGF | hepatoma-derived growth factor (high-mobility group protein 1-like) | 200896_x_at | 4.2 |
| HIF1A | hypoxia-inducible factor 1, alpha subunit (basic helix-loop-helix transcription factor) | 200989_at | 8.3 |
| IFNGR2 | interferon gamma receptor 2 (interferon gamma transducer 1) | 201642_at | 4.5 |
| IGF1 | insulin-like growth factor 1 (somatomedin C) | 209541_at | 7.6 |
| IGFBP3 | insulin-like growth factor binding protein 3 | 210095_s_at | 4.3 |
| IGFBP4 | insulin-like growth factor binding protein 4 | 201508_at | 5.8 |
| IGFBP5 | insulin-like growth factor binding protein 5 | 211959_at | 2.4 |
| IGFBP7 | insulin-like growth factor binding protein 7 | 201162_at | 5.4 |
| IL1R1 | interleukin 1 receptor, type I | 202948_at | 5.1 |
| ITGAV | integrin, alpha V (vitronectin receptor, alpha polypeptide, antigen CD51) | 202351_at | 10.1 |
| ITGB1 | integrin, beta 1 (fibronectin receptor, beta polypeptide, antigen CD29 includes MDF2, MSK12) | 211945_s_at | 6.1 |
| JAK1 | Janus kinase 1 (a protein tyrosine kinase) | 201648_at | 5 |
| JUN | v-jun sarcoma virus 17 oncogene homolog (avian) | 201464_x_at | 6 |
| KDELR2 | KDEL (Lys-Asp-Glu-Leu) endoplasmic reticulum protein retention receptor 2 | 200699_at | 4.9 |
| KDELR3 | KDEL (Lys-Asp-Glu-Leu) endoplasmic reticulum protein retention receptor 3 | 207265_s_at | 7.6 |
| LEPR, LEPROT | (leptin receptor, leptin receptor overlapping transcript) | 202377_at | 6.2 |
| LOC440847, ST13 | (similar to heat shock 70kD protein binding protein; progesterone receptor-associated p48 protein; putative tumor suppressor ST13; Hsp70-interacting protein; suppression of tumorigenicity 13 (colon carcinoma) (Hsp70-interacting protein) ..., suppression of tumorigenicity 13 (colon carcinoma) (Hsp70 interacting protein)) | 207040_s_at | 4.9 |
| LRP1 | low density lipoprotein-related protein 1 (alpha-2-macroglobulin receptor) | 200785_s_at | 6.1 |
| LRP10 | low density lipoprotein receptor-related protein 10 | 201412_at | 5 |
| LTBP1 | latent transforming growth factor beta binding protein 1 | 202729_s_at | 4.4 |
| LTBP2 | latent transforming growth factor beta binding protein 2 | 204682_at | 6.7 |
| NCOA4 | nuclear receptor coactivator 4 | 210774_s_at | 5.4 |
| NCOR2 | nuclear receptor co-repressor 2 | 207760_s_at | 4.8 |
| NGFRAP1 | nerve growth factor receptor (TNFRSF16) associated protein 1 | 217963_s_at | 5.5 |
| NR3C1 | nuclear receptor subfamily 3, group C, member 1 (glucocorticoid receptor) | 201865_x_at | 4.1 |
| PDGFC | platelet derived growth factor C | 218718_at | 7.7 |
| PDGFRA | platelet-derived growth factor receptor, alpha polypeptide | 203131_at | 4.4 |
| PDGFRB | platelet-derived growth factor receptor, beta polypeptide | 202273_at | 3.8 |
| PDGFRL | platelet-derived growth factor receptor-like | 205226_at | 3.9 |
| PGRMC1 | progesterone receptor membrane component 1 | 201121_s_at | 4.3 |
| PTEN | phosphatase and tensin homolog (mutated in multiple advanced cancers 1) | 225363_at | 4.4 |
| PTGFRN | prostaglandin F2 receptor negative regulator | 224937_at | 5.7 |
| PTPN11 | protein tyrosine phosphatase, non-receptor type 11 (Noonan syndrome 1) | 212610_at | 7.5 |
| PTPRO | protein tyrosine phosphatase, receptor type, O | 211600_at | 4.7 |
| RHOA | ras homolog gene family, member A | 200059_s_at | 5.6 |
| RPS6 | ribosomal protein S6 | 209134_s_at | 3.5 |
| SCARB2 | scavenger receptor class B, member 2 | 224983_at | 5.1 |
| SDFR1 | stromal cell derived factor receptor 1 | 202228_s_at | 6.9 |
| SHC1 | SHC (Src homology 2 domain containing) transforming protein 1 | 214853_s_at | 5.5 |
| SRPR | signal recognition particle receptor ('docking protein') | 200918_s_at | 4.5 |
| SRPRB | signal recognition particle receptor, B subunit | 218140_x_at | 6.2 |
| SSR1 | signal sequence receptor, alpha (translocon-associated protein alpha) | 200891_s_at | 7.8 |
| SSR2 | signal sequence receptor, beta (translocon-associated protein beta) | 200652_at | 4.1 |
| SSR4 | signal sequence receptor, delta (translocon-associated protein delta) | 201004_at | 4.7 |
| STAT1 | signal transducer and activator of transcription 1, 91kDa | 200887_s_at | 2.9 |
| STAT3 | signal transducer and activator of transcription 3 | 208991_at | 6.8 |
| STRAP | serine/threonine kinase receptor associated protein | 200870_at | 6.1 |
| TBL1XR1 | transducin (beta)-like 1X-linked receptor 1 | 223013_at | 4.8 |
| TGFB1I1 | transforming growth factor beta 1 induced transcript 1 | 209651_at | 6.7 |
| TGFB2 | transforming growth factor, beta 2 | 228121_at | 7.9 |
| TGFB3 | transforming growth factor, beta 3 | 209747_at | 4.9 |
| TGFBI | transforming growth factor, beta-induced, 68kDa | 201506_at | 3.5 |
| TGFBR1 | transforming growth factor, beta receptor I (activin A receptor type II-like kinase, 53kDa) | 224793_s_at | 5.3 |
| TGFBR2 | transforming growth factor, beta receptor II (70/80kDa) | 208944_at | 3.3 |
| TNFRSF1A | tumor necrosis factor receptor superfamily, member 1A | 207643_s_at | 3.9 |

Fold change analysis was performed using genes encoding known growth factors, receptors, cytokines, and proteins involved in the immune response and signaling pathways including angiogenesis and mTOR. Genes that were most differentially expressed between AF-set A and AF-set B are listed.
