# Supplementary material for: Identification of heterogeneity among soft tissue sarcomas by gene expression profiles from different tumors
Source: J Transl Med. 2008 May 6;6:23. doi: 10.1186/1479-5876-6-23 (PMC2412854; doi:10.1186/1479-5876-6-23)
Supplement: Additional file 3 — Genes upregulated 3-fold or more in MFH-A compared to MFH-B. Genes that were most differentially expressed between MFH-A and MFH-B were selected by e-Northern® analysis and are listed. [file 1479-5876-6-23-S3.doc]

Additional file 3. Genes upregulated 3-fold or more in MFH-A compared to MFH-B

| Fold Change (up in MFH-A) | Gene Symbol | Gene Name | Fragment Name |
| --- | --- | --- | --- |
| 16.1 | EFEMP1 | EGF-containing fibulin-like extracellular matrix protein 1 | 201842_s_at |
| 12.3 | ABI3BP | ABI gene family, member 3 (NESH) binding protein | 223395_at |
| 11.5 | IL8 | interleukin 8 | 202859_x_at |
| 10.6 | SEMA3C | sema domain, immunoglobulin domain (Ig), short basic domain, secreted, (semaphorin) 3C | 203789_s_at |
| 8.2 | GFPT2 | glutamine-fructose-6-phosphate transaminase 2 | 205100_at |
| 7.5 | CAV1 | caveolin 1, caveolae protein, 22kDa | 203065_s_at |
| 6.1 | CCL2 | chemokine (C-C motif) ligand 2 | 216598_s_at |
| 6 | AXL | AXL receptor tyrosine kinase | 202686_s_at |
| 5.9 | SLC2A14, SLC2A3 | solute carrier family 2 (facilitated glucose transporter), member 14, solute carrier family 2 (facilitated glucose transporter), member 3 | 202497_x_at |
| 5.3 | VEGF | vascular endothelial growth factor | 210512_s_at |
| 5.3 | SERPINE1 | serpin peptidase inhibitor, clade E (nexin, plasminogen activator inhibitor type 1), member 1 | 202628_s_at |
| 4.8 | TGFBR3 | transforming growth factor, beta receptor III (betaglycan, 300kDa) | 226625_at |
| 4.7 | SLC39A14 | solute carrier family 39 (zinc transporter), member 14 | 212110_at |
| 4.6 | NUCKS1 | nuclear casein kinase and cyclin-dependent kinase substrate 1 | 226880_at |
| 4.6 | IER3 | immediate early response 3 | 201631_s_at |
| 4.5 | MT1E | metallothionein 1E (functional) | 212859_x_at |
| 4.5 | BNIP3L, LOC440258 | BCL2/adenovirus E1B 19kDa interacting protein 3-like, similar to p40 | 221478_at |
| 4.5 | NDRG1 | N-myc downstream regulated gene 1 | 200632_s_at |
| 4.4 | ANTXR2 | anthrax toxin receptor 2 | 225524_at |
| 4.4 | SRPX | sushi-repeat-containing protein, X-linked | 204955_at |
| 4.4 | MMD | monocyte to macrophage differentiation-associated | 203414_at |
| 4.4 | LOC440737 | similar to 60S ribosomal protein L35 | 211456_x_at |
| 4.3 | CALU | calumenin | 200755_s_at |
| 4.2 | MT1H | metallothionein 1H | 206461_x_at |
| 4.2 | NOTCH2 | Notch homolog 2 (Drosophila) | 212377_s_at |
| 4 | MIF | macrophage migration inhibitory factor (glycosylation-inhibiting factor) | 217871_s_at |
| 4 | DPYSL2 | dihydropyrimidinase-like 2 | 200762_at |
| 3.9 | MT1E, MT1F | metallothionein 1E (functional), metallothionein 1F (functional) | 217165_x_at |
| 3.9 | IL6ST | interleukin 6 signal transducer (gp130, oncostatin M receptor) | 212195_at |
| 3.9 | FEZ2 | fasciculation and elongation protein zeta 2 (zygin II) | 215000_s_at |
| 3.9 | CSPG2 | chondroitin sulfate proteoglycan 2 (versican) | 211571_s_at |
| 3.8 | REXO2 | REX2, RNA exonuclease 2 homolog (S. cerevisiae) | 218194_at |
| 3.8 | GAS5 | growth arrest-specific 5 | 224841_x_at |
| 3.8 | PEA15 | phosphoprotein enriched in astrocytes 15 | 200788_s_at |
| 3.7 | NGFRAP1 | nerve growth factor receptor (TNFRSF16) associated protein 1 | 217963_s_at |
| 3.7 | MT2A | metallothionein 2A | 212185_x_at |
| 3.7 | RND3 | Rho family GTPase 3 | 212724_at |
| 3.7 | EIF1 | eukaryotic translation initiation factor 1 | 212227_x_at |
| 3.7 | PLSCR1 | phospholipid scramblase 1 | 202446_s_at |
| 3.7 | UBE2S, UBE2SP2 | ubiquitin-conjugating enzyme E2S, ubiquitin-conjugating enzyme E2S pseudogene 2 | 202779_s_at |
| 3.7 | PAM | peptidylglycine alpha-amidating monooxygenase | 202336_s_at |
| 3.7 | UGCG | UDP-glucose ceramide glucosyltransferase | 224967_at |
| 3.7 | SOD2 | superoxide dismutase 2, mitochondrial | 221477_s_at |
| 3.7 | PPM1J, RHOC | protein phosphatase 1J (PP2C domain containing), ras homolog gene family, member C | 200885_at |
| 3.7 | GNG12 | guanine nucleotide binding protein (G protein), gamma 12 | 212294_at |
| 3.7 | SLCO2B1 | solute carrier organic anion transporter family, member 2B1 | 203473_at |
| 3.7 | ADAM10 | ADAM metallopeptidase domain 10 | 202603_at |
| 3.7 | XBP1 | X-box binding protein 1 | 200670_at |
| 3.6 | TGFBR2 | transforming growth factor, beta receptor II (70/80kDa) | 208944_at |
| 3.6 | STAT1 | signal transducer and activator of transcription 1, 91kDa | 200887_s_at |
| 3.6 | DAB2 | disabled homolog 2, mitogen-responsive phosphoprotein (Drosophila) | 201279_s_at |
| 3.6 | ABL1 | v-abl Abelson murine leukemia viral oncogene homolog 1 | 202123_s_at |
| 3.5 | PGRMC1 | progesterone receptor membrane component 1 | 201120_s_at |
| 3.5 | C1QR1 | complement component 1, q subcomponent, receptor 1 | 202878_s_at |
| 3.5 | RPL22 | ribosomal protein L22 | 221726_at |
| 3.5 | SF3B14 | splicing factor 3B, 14 kDa subunit | 223416_at |
| 3.5 | LOC440847, ST13 | similar to heat shock 70kD protein binding protein; progesterone receptor-associated p48 protein; putative tumor suppressor ST13; Hsp70-interacting protein | 207040_s_at |
| 3.5 | MT1G | metallothionein 1G | 204745_x_at |
| 3.5 | CLTA | clathrin, light polypeptide (Lca) | 216295_s_at |
| 3.5 | FBL | fibrillarin | 211623_s_at |
| 3.5 | ANP32B | acidic (leucine-rich) nuclear phosphoprotein 32 family, member B | 201306_s_at |
| 3.4 | RPL15 | ribosomal protein L15 | 221476_s_at |
| 3.4 | MT1X | metallothionein 1X | 208581_x_at |
| 3.4 | LOC389168, SET | SET translocation (myeloid leukemia-associated), similar to SET protein (Phosphatase 2A inhibitor I2PP2A) (I-2PP2A) (Template activating factor I) (TAF-I) (HLA-DR associated protein II) (PHAPII) (Inhibitor of granzyme A-activated DNase) (IGAAD) | 213047_x_at |
| 3.4 | ENO1, LOC400733 | enolase 1, (alpha), hypothetical LOC400733 | 217294_s_at |
| 3.3 | UGP2 | UDP-glucose pyrophosphorylase 2 | 205480_s_at |
| 3.3 | SFRS7 | splicing factor, arginine/serine-rich 7, 35kDa | 214141_x_at |
| 3.3 | RPL14, RPL14L | ribosomal protein L14, ribosomal protein L14-like | 200074_s_at |
| 3.3 | FGG | fibrinogen gamma chain | 226621_at |
| 3.3 | RALA | v-ral simian leukemia viral oncogene homolog A (ras related) | 224880_at |
| 3.2 | CEBPB | CCAAT/enhancer binding protein (C/EBP), beta | 212501_at |
| 3.2 | EIF3S7 | eukaryotic translation initiation factor 3, subunit 7 zeta, 66/67kDa | 200005_at |
| 3.2 | PSMA1 | proteasome (prosome, macropain) subunit, alpha type, 1 | 201676_x_at |
| 3.2 | DDR2 | discoidin domain receptor family, member 2 | 225442_at |
| 3.2 | TMED10 | transmembrane emp24-like trafficking protein 10 (yeast) | 212352_s_at |
| 3.2 | DAZAP2 | DAZ associated protein 2 | 200794_x_at |
| 3.1 | ANXA5 | annexin A5 | 200782_at |
| 3.1 | CTSL, CTSLL3, HCTSL-s | cathepsin L, cathepsin L-like 3, cathepsin L-like protein | 202087_s_at |
| 3.1 | RPL36AL | ribosomal protein L36a-like | 207585_s_at |
| 3.1 | SPCS2 | signal peptidase complex subunit 2 homolog (S. cerevisiae) | 201240_s_at |
| 3.1 | SLC38A2 | solute carrier family 38, member 2 | 220924_s_at |
| 3.1 | MXRA7, PSMD14 | matrix-remodelling associated 7, proteasome (prosome, macropain) 26S subunit, non-ATPase, 14 | 212509_s_at |
| 3.1 | DHX15 | DEAH (Asp-Glu-Ala-His) box polypeptide 15 | 201385_at |
| 3.1 | ARHGAP1 | Rho GTPase activating protein 1 | 202117_at |
| 3.1 | RBPSUH | recombining binding protein suppressor of hairless (Drosophila) | 211974_x_at |
| 3.1 | APEX1 | APEX nuclease (multifunctional DNA repair enzyme) 1 | 210027_s_at |
| 3.1 | ATP2A2 | ATPase, Ca++ transporting, cardiac muscle, slow twitch 2 | 209186_at |
| 3.1 | LIPA | lipase A, lysosomal acid, cholesterol esterase (Wolman disease) | 201847_at |
| 3 | MATR3 | matrin 3 | 214363_s_at |

Fold change analysis was performed comparing gene expression of MFH-A to MFH-B samples. Genes that were most differentially expressed between MFH-A and MFH-B were selected by e-Northern® analysis and are listed.
